# Study on the Anticondensation Characteristics of Liquid Silicone Rubber Temperature-Control Coatings

**DOI:** 10.3390/polym11081282

**Published:** 2019-08-01

**Authors:** Juyi Guo, Xilin Wang, Jun Wang, Chuan Chen, Yu Liu, Weinan Fan, Zhidong Jia

**Affiliations:** 1 Engineering Laboratory of Power Equipment Reliability in Complicated Coastal Environment, Graduate school at Shenzhen, Tsinghua University, Shenzhen 518055, China; 2 China National Electric Apparatus Research Institute Co., Ltd., Guangzhou 510080, China; 3 Guangzhou Power Supply Bureau Co., Ltd., Guangzhou 510600, China

**Keywords:** condensation, liquid silicone rubber, phase change materials, phase change capsule, ring-network cabinet

## Abstract

Metal cabinets such as switch cabinets and ring network cabinets have the advantages of small footprints and good protection for equipment and offer neat and orderly protection. They are widely used in power systems. In a hot and humid environment, condensation can easily cause equipment to age and even cause insulation failure. Therefore, research on reliable anticondensation methods is of great significance for the safe operation of power equipment. In this study, phase change capsules with phase transition temperatures of 22 and 32 °C were used as fillers and liquid silicone rubber was used as a matrix to prepare liquid silicone rubber composites filled with phase change capsules for a temperature-control coating. Studies have shown that liquid silicone rubber coatings containing phase change capsules can significantly enhance the anticondensation performance of metal cabinets. By using a temperature-control coating on the surface where the cabinet experiences condensation, the temperature difference between the surface and the dew point is reduced, thereby slowing down the condensation rate and even preventing condensation.

## 1. Introduction

**Figure 1:**
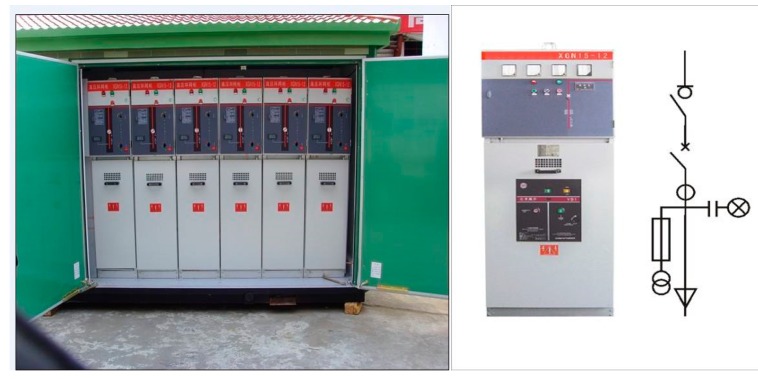
Ring network cabinet structure diagram (left is a physical image, and right is a function diagram).

Ring network cabinets are devices for placing high-voltage power equipment (mainly high-voltage switchgear) in a metal or nonmetal insulated cabinet with load switches and fuses, which can be seen in Figure 1. As compact power equipment, the ring network cabinet has a load switch, circuit breaker, protection equipment, operation power supply, etc., and has the advantages of small space, simple structure and high safety [1].

Condensation can occur when the humidity inside a ring network cabinet placed in a high-humidity environment is increased due to the intrusion of water vapor [2]. When the wall temperature of the cabinet is lower than the dew point temperature, condensation occurs [3]. Metal cabinets in high temperature and high humidity and low temperature and high humidity environments with insufficient internal heat dissipation and dehumidification capacity experience a variety of failures when condensation occurs: (1) cable outlet cable breaks; (2) liquid volume shortening the creepage distance to triggers three phases of short circuit; (3) mechanism corrosion, resulting in viscous operation, and even in direct rust making the cabinet inoperable; (4) direct breakdown inside the circuit breaker causing a short circuit ground fault; (5) insulation separator absorbing water vapor and dust, causing creepage and reducing insulation strength [4].

In power equipment, there are many possible reasons for condensation: (1) a cable trench is located under the cabinet; if the seal is not tight, water vapor may invade the cabinet, and the cable trench is generally hot due to running heat. When the surface is below the dew point temperature, condensation will occur; (2) The position where the cabinet is placed is underground, which is a location that is difficult to ventilate and dehumidify. The long-term operation humidity will gradually increase, water vapor will accumulate continuously, and the relative humidity will reach 100%, which will cause condensation; (3) The cabinet heats up, but the external temperature drops rapidly. The heat conduction speed of the cabinet wall as a metal is very fast, which lowers the air dew point temperature below that of the cabinet, and condensation occurs; (4) The insulating spacer material has a certain ability to adsorb water vapor. If the temperature inside the cabinet is always higher than the external temperature, condensation can be prevented [5]. Therefore, it is of great significance to study measures to improve the anti-condensation ability of these cabinets and reduce the occurrence of condensation without power failure.

For the condensation phenomenon of metal cabinets, there are three protection ideas for controlling the temperature, relative humidity and absolute humidity. The principle of controlling the relative humidity method is mainly to heat the humid air [6,7]. The absolute humidity control method is mainly used to reduce the humidity inside the cabinet by ventilation and moisture absorption [8,9,10].

In the hot and humid environment, because of the limited cooling and dehumidification capacity, condensation may occur due to changes in temperature and humidity, which may cause serious damage to the insulation inside the cabinet. Existing methods such as dehumidifiers, heaters, ventilation devices, moisture-proof agents, and hydrophobic coatings have limitations. Therefore, it is important to find ways to prevent green condensation, high reliability, and easy maintenance. Compared with the previous method, the surface coating method has the advantages of being clean, simple and convenient and not consuming energy [11]. Phase change materials (PCMs) are materials that absorb or release large amounts of heat through a phase change process at a constant temperature [12,13]. In this paper, a composite material of a phase change capsule and liquid silicone rubber was prepared and used as a temperature-control coating to reduce condensation on the cabinet wall and explore the mechanism of the coating to slow down condensation.

## 2. Materials and Methods

### 2.1. Material Processing and Sample Preparation

The composite material with different fillers was prepared as follows:

Liquid silicone rubber was from Shenzhen Square Silicone Co., Ltd. (Shenzhen, China), the phase change material (phase transition temperature 22 °C phase change composite material, phase transition temperature 32 °C phase change composite material, using silica aerogel as wall material adsorption core material, the core material is paraffin) was from Hubei Semer New Energy Technology Co., Ltd. (Wuhan, China) with a particle size of approximately 200 nm. The phase change temperature of the 22 °C phase change material is referred to as F1, and the phase change temperature of 32 °C is F2. Preparation ratios are shown in Table 1:

The wall surface of the ring network cabinet was simulated using a stainless-steel surface of 6 cm × 6 cm × 0.03 cm. The coating process consisted of the following steps:

(1) The surface of the test piece was cleaned with a cloth moistened with absolute ethanol;

(2) Due to the weak adhesion of liquid silicone rubber, it was necessary to use a coupling agent to enhance the surface adhesion. The coating agent KH-560 (γ-(2,3-epoxypropoxy) propyltrimethoxysilane) can be applied by spraying or brushing. The surface was evenly adhered with KH-560, and no droplets were accumulated. Then, the test piece coated with the coupling agent was evaporated at 80 °C to dissolve the coupling agent solvent. A uniform translucent coupling agent layer was formed on the surface;

(3) The two-component liquid silicone rubber was mixed uniformly in a 1:1 ratio. The two components were SSR1501-40MA and SSR1501-40MB, purchased from Shenzhen Square Silicone Co., Ltd. (Shenzhen, China). To make the coating surface flat, the prepared liquid silicone rubber was added to D4 silicone oil (Octamethylcyclotetrasiloxane) in a certain proportion. The coating was prepared to meet the coating requirements and stirred at a stirring speed of 1000 rpm for 30 min. Then it was allowed to stand until the coating was free of bubbles;

(4) After the coating agent was coated, the surface of the test piece was coated with a liquid glue coating with a fine brush. After the coating was completed, the bubbles were discharged in a vacuum environment, and then, the test piece was placed in an environment of 80 °C for 30 min. The coating amount was determined to be 0.032 g/cm^2^, and the thickness was 0.3 mm according to the surface tension nonoverflow edge as a standard and with reference to the relevant coating thickness standard.

### 2.2. Characterization

To observe the microscopic morphology of the sample and the powder, scanning electron microscopy (SEM, Hitachi SU8010, Tokyo, Japan) was used for observation. All the images were acquired under 5 kV voltage. All the samples had already been sputtered with gold and evacuated in 20 MPa for 30 min. 

### 2.3. Test System and Experimental Details

Thermogravimetric analysis (TG or TGA) refers to a test method for measuring material weight loss and endothermic power at programmed temperatures. The test uses a thermogravimetric analyzer (Shanghai, China) with a test temperature range of 0 to 1000 °C and a heating rate of 5 °C/min.

DSC measures the difference in power input to the sample and reference as a function of temperature over time. It takes the rate of the endothermic or exothermic heat of the sample, that is, the heat flow rate dH/dt (in milliwatts) as the ordinate and the temperature T or time t as the abscissa, and can measure thermodynamic and kinetic parameters, such as specific heat, reaction heat, heat of transformation, etc. The DSC method was used to determine the characteristics of the sample and the powder, and the power curve was obtained. The thermal transition characteristics of the material were characterized by the mass flow rate per unit mass (mW/mg), and the transformation temperature, specific heat and enthalpy change were further analyzed.

To study the thermal conductivity of the composite material, thermal conductivity measurements were performed using a flash thermal conductivity meter (Netzsch LFA447. Selb, Germany) and the experiment was carried out according to the relevant standards of ASTM E1461.

**Figure 2:**
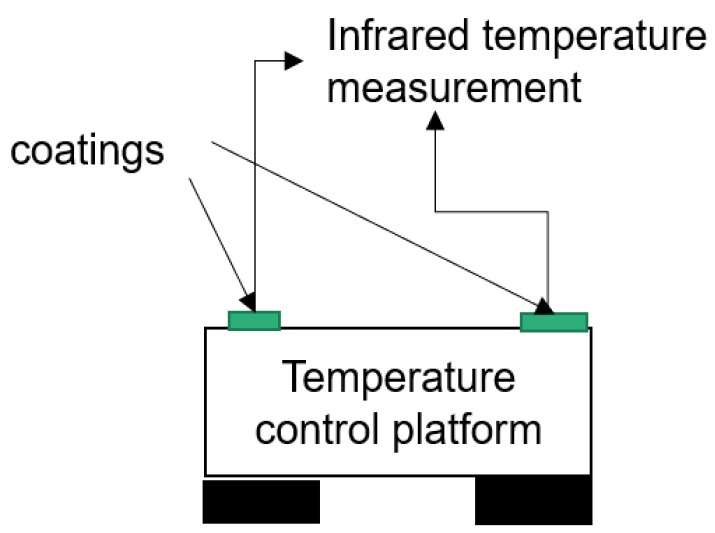
Temperature control test platform.

To explore the thermal conductivity of the coating during the specific heat transfer process, the designed testing platform is shown in Figure 2. The temperature control platform can set a certain temperature, and the temperature of the coating surface is monitored by infrared to explore the temperature characteristics of the coating during heating and cooling.

**Figure 3:**
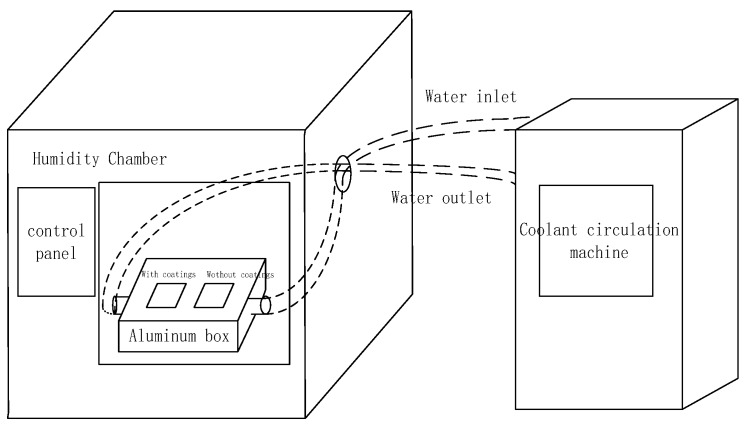
Conceptual diagram of the simulation experiment device.

To simulate the actual environment of condensation, it is necessary to control three variables: wall temperature, ambient temperature and ambient humidity. Then, the settings were changed to simulate different working conditions. Specifically, 30 cm wide, 15 cm high, and 6 cm long aluminum closed chambers with metal pipes in and out of the water were made. The sample was placed on the surface of the cavity. The temperature of the aluminum was controlled by controlling the water temperature to control the temperature of the sample. The entire device was placed into a constant temperature and humidity chamber to simulate the external temperature and humidity environment. The inlet and outlet pipes of the temperature-controlled aluminum box were introduced in the side vents of the box, and the other parts were filled with heat-insulating foam to ensure that there was no heat exchange between the hot and humid air in the incubator and the outside. The surface temperature of the coated sample was the same as that of the constant temperature and humidity chamber before the start of the experiment. At the beginning of the experiment, the coated samples were placed on the surface of the aluminum box to simulate the contact between the low temperature cabinet wall and the coating. Figure 3 is a conceptual diagram of the simulation experiment device.

The working conditions were set as shown in the first entry in Table 2 to explore the effects of different temperatures, different relative humidity values, and different dew point temperature differences on the anticondensation time of different coatings.

The moisture test strip WT was used to test for condensation. The test paper turns from orange to yellow after touching water. The test paper was cut into small pieces of 1 cm × 1 cm and placed on the surface of the liquid silicone rubber coating with tweezers, and one piece was placed at each of the four corners and the center. When the five pieces were discolored, it was judged that the test pieces showed condensation.

## 3. Results

### 3.1. Characterization (SEM) Results

**Figure 4:**
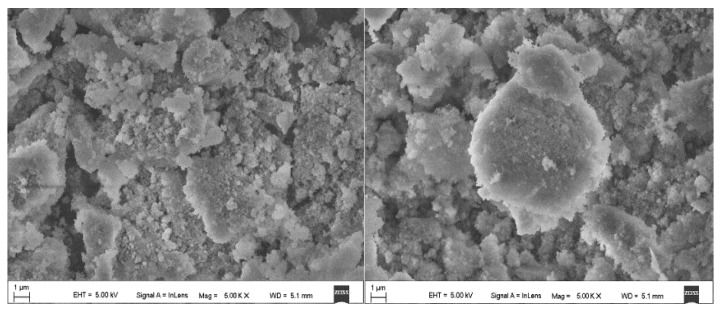
SEM image of F1 (**left**) and F2 (**right**) (5000× magnification).

**Figure 5:**
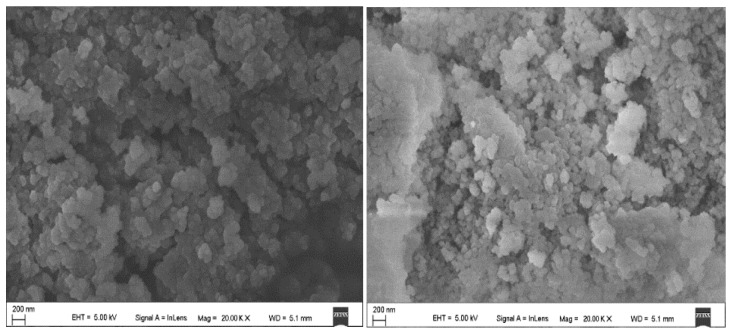
SEM image of F1 (**left**) and F2 (**right**) (20,000× magnification).

First, the powder of the two-phase change microcapsules was observed, as shown in Figure 4 (5000× magnification) and Figure 5 (20,000× magnification). The two powders were similar, and the adsorbed paraffin had a particle size of approximately 200 nm. Additionally, there was a certain agglomeration phenomenon.

### 3.2. Analysis of the Thermal Weight Loss Characteristics of the Composite Coatings

**Figure 6:**
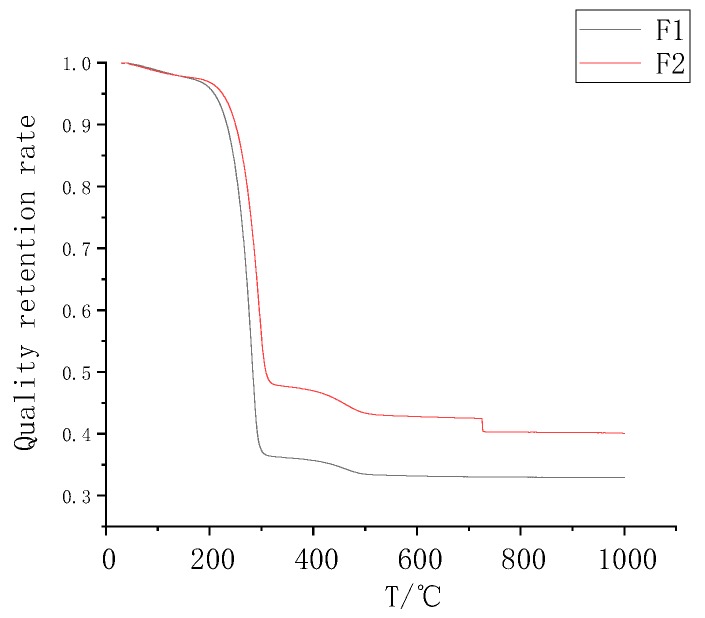
F1 andF2 TGA diagrams.

Figure 6 is a thermogravimetric image of powders F1 and F2. As shown in Figure 6, the lower part is the thermogravimetric image of F1, and the upper part is the thermogravimetric image of F2. The powder could maintain stability under 300 °C. The boiling point of paraffin was reached at 300 °C, resulting in a rapid decrease in mass. The quality loss trends of the two powders were basically the same, and the starting points of the significant changes in the mass loss rate were basically the same. The difference was the difference in the mass loss ratio. The chemical composition of the two powders was similar; the difference was only the difference in the proportion of the specific components, that is, the difference in the proportion of paraffin. Silica has good thermal stability and does not substantially decompose below 1000 °C.

**Figure 7:**
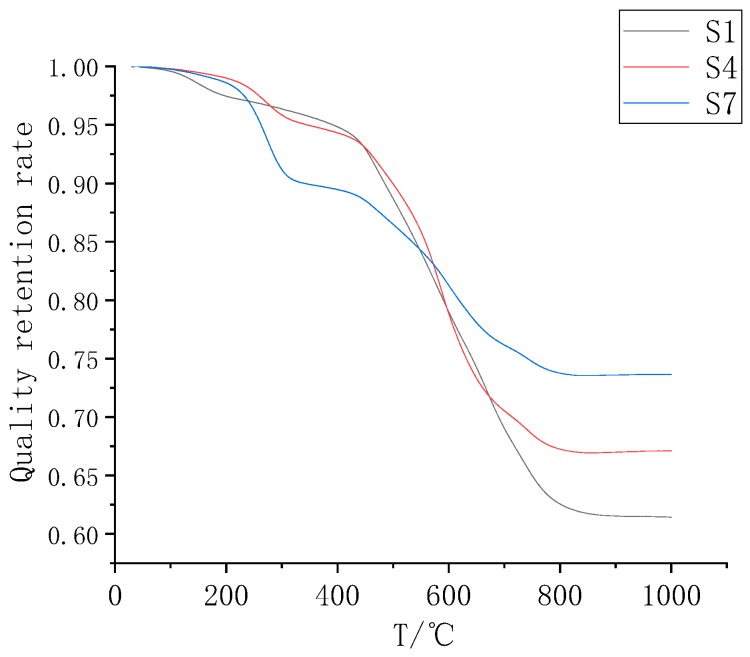
S1, S4, and S7 TGA diagrams.

Figure 7 is a graph showing the thermal weight loss of the S1, S4, and S7 samples, from bottom to top S1, S4, and S7. Observing the mass loss curve of the F1 filled sample, it can be found that the filled samples S4 and S7 had obvious mass loss rate changes at approximately 300 °C. Compared with the above figure, it can be found that the curve change was caused by the rapid thermal decomposition of F1 powder. The F1 mass fraction in S7 was higher, so the mass loss rate change at approximately 300 °C was larger than that of S4. In terms of thermal stability, powder addition could also increase the thermal stability of the composite as a whole under 1000 °C. For the mass loss rate at 1000 °C, the S4 and S7 samples had significant reductions relative to the unadded sample S1.

**Figure 8:**
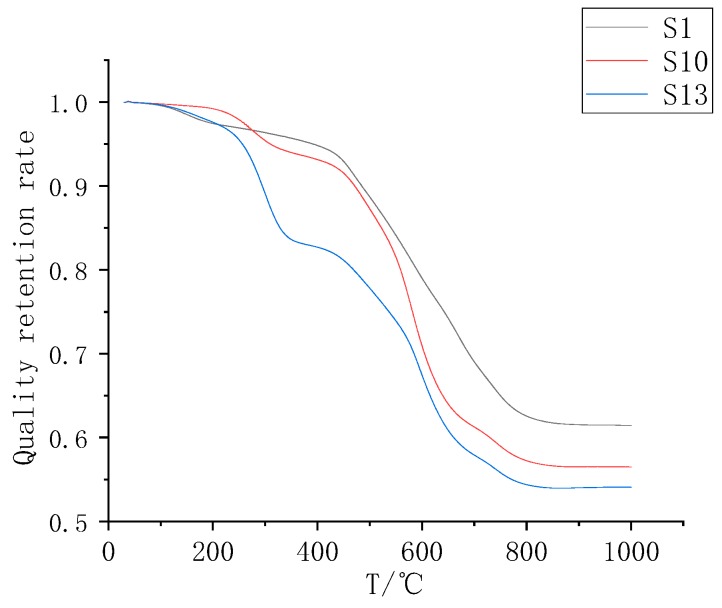
S1, S10, and S13 TGA diagrams.

Figure 8 is a graph showing the thermal weight loss of the S1, S10, and S13 samples. Comparing the mass loss curves of the F2 filled samples, it can be found that at 300 °C, the filled samples S10 and S13 had obvious changes in the mass loss rate, and the change rules were the same as those of the F1 filled samples. The F2 mass fraction of S13 was higher, so the mass loss rate change at approximately 300 °C was larger than that of S10. In terms of thermal stability, F2 powder addition reduced the thermal stability of the composite as a whole under 1000 °C. For the mass loss rate at 1000 °C, the S4 and S7 samples had a significant increase relative to that of the unadded sample S1. Because the F2 powder was present in a higher content than the F1 paraffin, the mass loss caused by decomposition was greater.

### 3.3. Study on the Thermal Storage Properties of Composite Coatings

**Figure 9:**
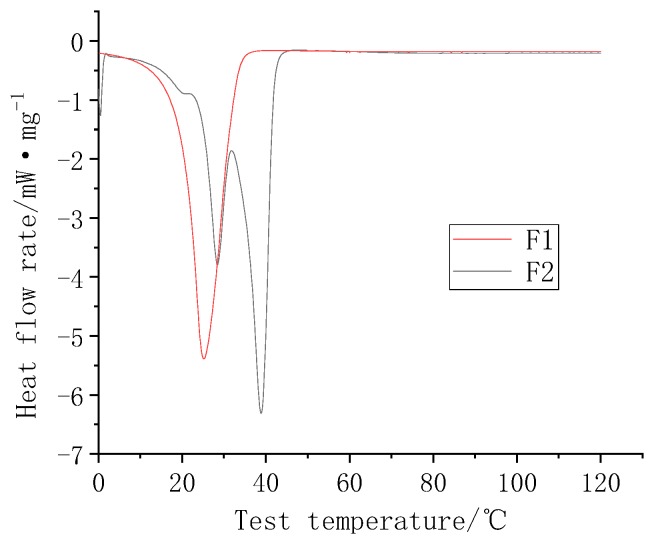
F1 and F2 DSC diagrams.

The DSC curves of the two powders are shown in Figure 9. The phase transition temperature of F1 was approximately 22 °C, and F2 had two different phase transition points at 24 and 38 °C because the paraffin in F2 was a mixture. The melting point was over a range, and the two carbon chain lengths were different [14]. The paraffin melted at two different temperatures. The F1 and F2 phase change heat storage values were 70.5 and 76.7 J/g, respectively.

**Figure 10:**
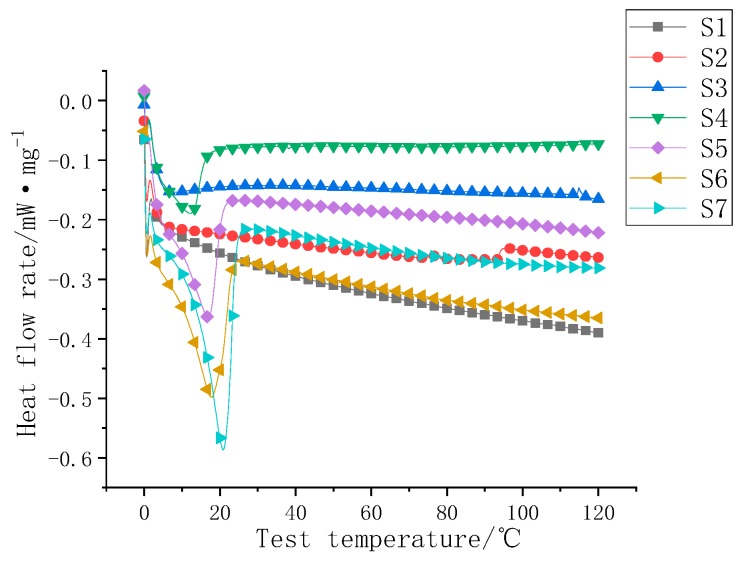
S1–S7 DSC diagrams.

The DSC curves of S1–S7 are shown in Figure 10.

S1 is an unfilled sample. It did not show a significant heat flow inflection point from 0 to 120 °C. It did not have obvious absorption and release characteristics at room temperature. Number two was the 2.5% F1-doped sample, and there was no obvious heat flow inflection point. The mass fraction of F1 in the S3–S7 sample gradually increased; the inflection point of the heat flow rate diagram gradually increased; and the sample of S7 was close to the phase transition temperature point of the powder (22 °C). The phase change enthalpy of the apparent phase change sample (S3–S7) was analyzed, and the endothermic heat of the liquid silicone rubber was removed by curve fitting. The endothermic heat of the phase change filler was simply calculated. The heat flow inflection point and phase change heat storage are listed in Table 3.

**Figure 11:**
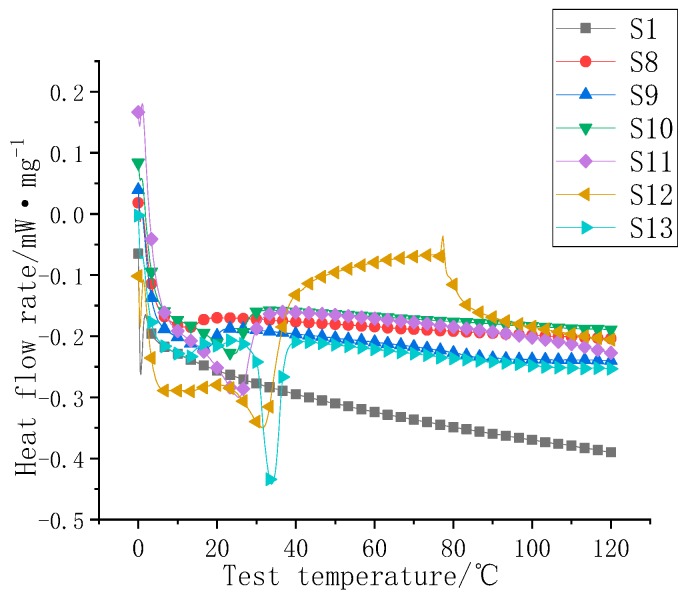
S1 and S8–S13 DSC diagrams.

The DSC curves of S8–S13 are shown in Figure 11, and S1 was used as a control. First, the heat flow rate map was obtained from the DSC chart. As shown in Figure 11, S8 was the 2.5% F2-doped sample, which had a very high phase transition temperature of 14.1 °C. The mass fraction of F2 in the S8–S13 sample gradually increased, and the inflection point of the heat flow rate diagram, that is, the phase transition point, gradually increased. The sample of S13 was close to the phase transition temperature point of the powder (32 °C). The heat flow inflection point and heat storage are shown in Table 4.

### 3.4. Testing Composite Coating Thermal Conductivity

**Figure 12:**
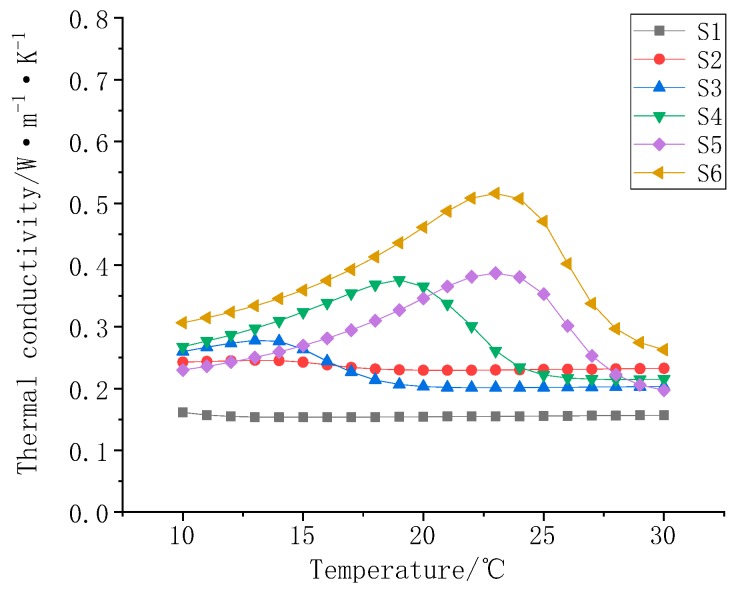
Thermal conductivity of S1–S7.

Figure 12 is a graph of the thermal conductivity of S1–S7 as a function of temperature. Comparing the thermal conductivity curves of the samples, it can be seen that the thermal conductivity of the composites gradually increases as the filler mass fraction increases. A sharp rise in thermal conductivity occurs in the phase transition temperature range of the composite material, and the increase can reach more than 50%.

**Figure 13:**
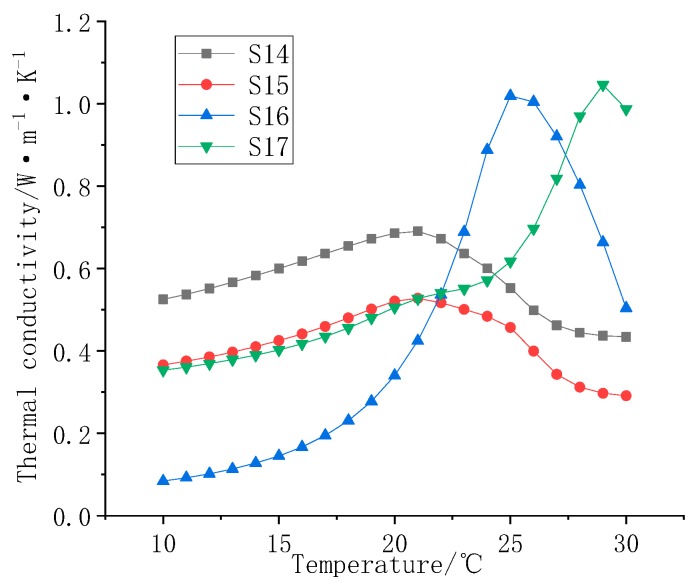
Thermal conductivity of S1 and S8–S13.

Figure 13 is a graph of the thermal conductivity of S1 and S8–S13 as a function of temperature. Similar to the F1 filled samples, the F2 filled samples also show a sharp rise near the phase transition temperature. Compared with the F1 filled samples, the F2 filled samples had a slightly lower thermal conductivity, and the thermal conductivity decreases with increasing filler ratio. Because the phase transition temperature of the F2 filled sample is higher, the temperature range corresponding to the steep rise zone is also higher.

### 3.5. Study on the Temperature Control Performance of Composite Coatings

**Figure 14:**
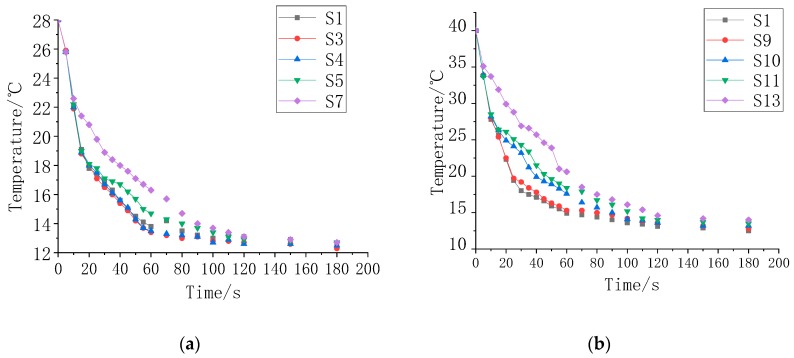
Temperature control performance of the composite coatings, (**a**) sample S1, S3, S4, S5 and S7; (**b**) sample S1, S9, S10, S11 and S13.

Figure 14a is a graph showing the decrease in the coating temperature from 28 °C overtime when the temperature of the temperature control platform is 8 °C, and Figure 14b is an image of the coating temperature decreasing from 40 °C over time when the temperature control platform was 8 °C. As the phase transition temperature of the composite coating increases, the "platform" temperature also rose, and the slower the temperature change in the phase change interval was, the stronger the heat storage capacity of the coating. As the mass fraction of fillers F1 and F2 increased, the heat storage capacity of the composite coating also increased, and the temperature control time was longer. Outside the phase transition temperature range, the temperature profiles of the coatings were basically the same, which means that the temperature control characteristics mainly depended on the heat storage in the phase transition temperature range.

### 3.6. The Results of the Condensation Simulation Experiment

**Figure 15:**
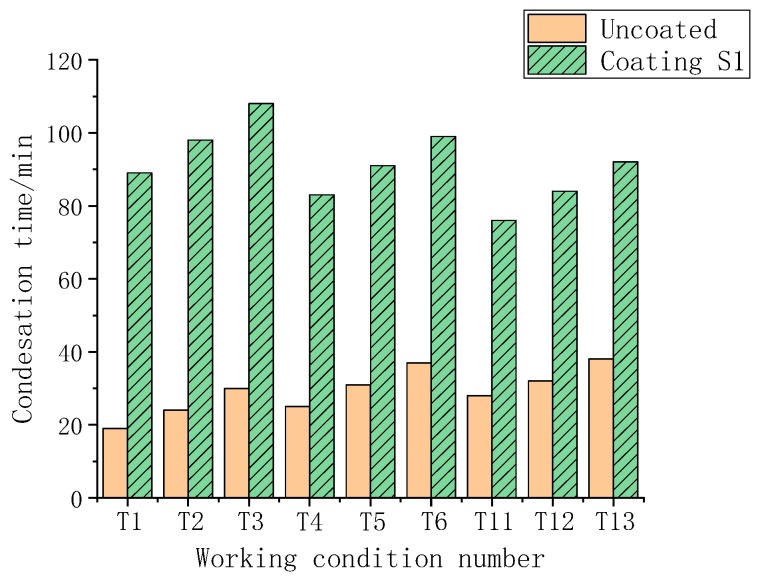
Coating’s effect on the anticondensation time.

The influence of the presence or absence of the coating on the occurrence time of condensation is compared and analyzed in Figure 15.

According to the analysis, under any working condition, the time of occurrence of condensation on the coated sample was longer than that on the uncoated, and the visible coating had a significant effect on the anticondensation behavior. The reason for this result is mainly because the thermal conductivity of the coating was significantly lower than the thermal conductivity of the steel sheet, and the surface temperature was lowered more slowly, which increased the time until condensation. In the lateral comparison between groups (T1–T3, T4–T6, and T11–T13), itwas found that at the same temperature and dew point temperature difference, the higher the humidity was, the more likely it was that condensation occurs. Compared with the groups with different humidity (T1, T4, T11; T2, T5, T12; and T3, T6, T13), when the temperature was the same as the dew point temperature, the higher the temperature was, the more likely it was that condensation would occur.

**Figure 16:**
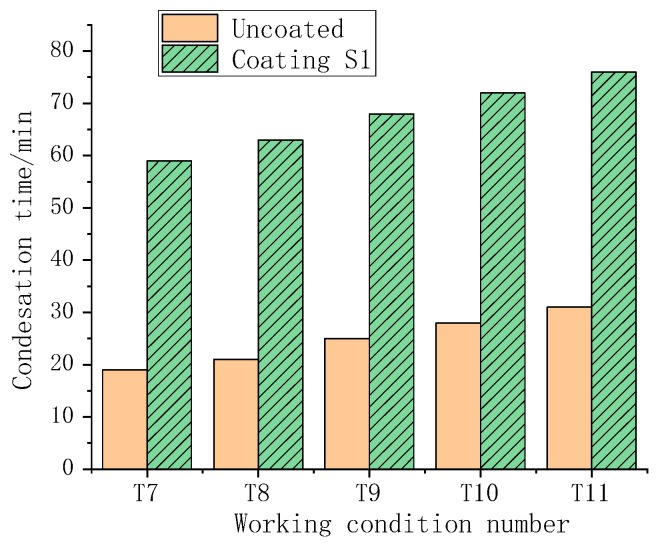
Effect of different dew point temperature differences on the occurrence time of condensation.

According to the working condition T7–T11, the influence of the dew point temperature difference on the occurrence time of condensation was investigated, as shown in Figure 16.

As shown in Figure 16, when the dew point temperature difference was increased, the condensation occurrence time was shortened, and condensation occurred earlier. This is true for samples with or without the coating. When the temperature difference of the dew point became larger, the heat transfer rate between the wet air and the surface increased, so surface condensation was more likely to occur.

**Figure 17:**
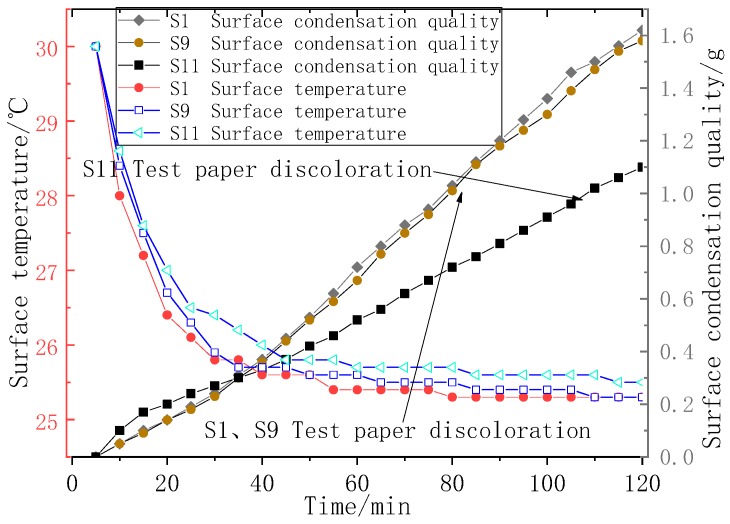
S1, S9, and S11 condensation process diagram under working condition T11.

The influence of each coating on the anticondensation performance of working condition T11 is shown in Figure 17. The surface condensation quality of the coating surface and the surface temperature were measured in real time, and the behavior of different coatings during the appearance of condensation was analyzed.

As shown in Figure 17, the ambient temperature of T11 was 30 °C, the surface temperature was 22 °C, the phase transition temperature of coating S9 was 19.2 °C, and the phase transition temperature of S11 was 26.2 °C. It can be seen that in the phase transition temperature range of coating S11, the temperature platform of the coating appeared, and the temperature drop was slower than that of S1 and S9. Additionally, the temperature difference of the dew point was reduced. From the point of view of the condensation rate, a smaller temperature difference represents a smaller condensation rate, and the coating growth rate of coating S11 was slower than that of S9 and S1. When the amount of condensation reached approximately 1 g, the test paper was completely discolored, and it was judged that the coating had condensation. When the temperature was near the phase transition temperature, the coating could effectively slow the temperature change, thereby reducing the condensation speed by reducing the dew point temperature difference.

**Figure 18:**
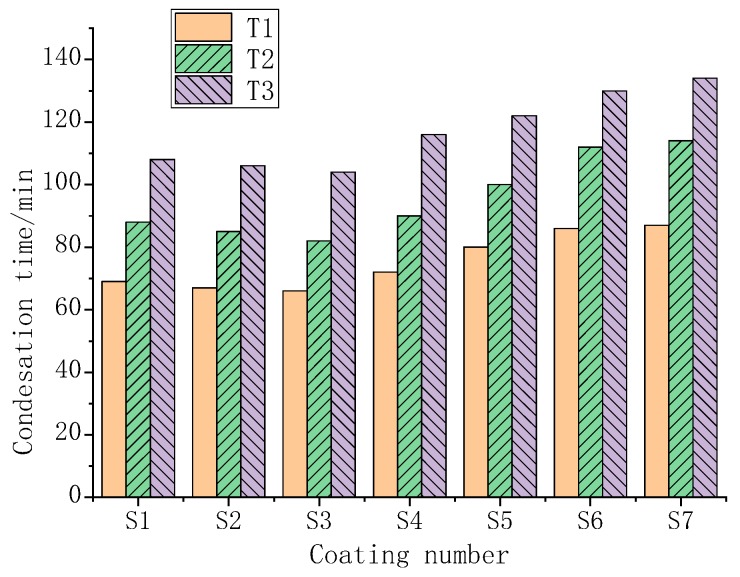
Different coating condensation times under three working conditions at 20 °C.

The anticondensation time of the coating was studied under three working conditions at a temperature of 20 °C, as shown in Figure 18. T1–T3 were working conditions with a temperature of 20 °C. The surface temperature of T1 was set to 14.3 °C, the surface temperature of T2 was 12.4 °C, and the surface temperature of T3 was set to be 10.4 °C. The corresponding dew point temperature was set to maintain a 4 °C difference.

As shown in Figure 18, the coatings S1 and S2 have no obvious phase change characteristics, and the S3 phase transition temperature is not in the range of the surface temperature and dew point temperature. There was no obvious difference in the anticondensation time among S1, S2 and S3. The phase transition temperatures of S4, S5, S6, and S7 show obvious anticondensation performance in the interval between the surface temperature and dew point temperature. The phase change heat storage increases with increasing serial number, and the improvement in the anticondensation performance also increases as the heat storage increases.

**Figure 19:**
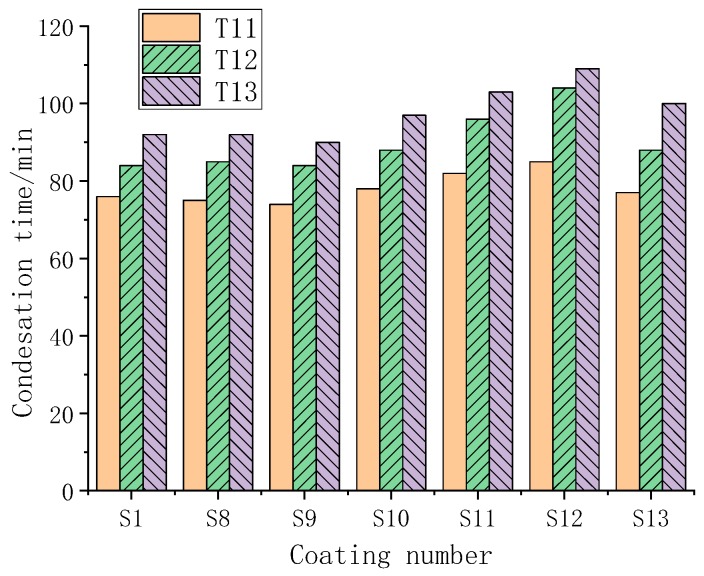
Different coating condensation times under three working conditions at 30 °C.

The condensation occurrence time of the coating was studied under three working conditions at a temperature of 30 °C, as shown in Figure 19.

As shown in Figure 19, T11–T13 were the working conditions at a temperature of 30 °C, a dew point temperature of 18 °C, and a surface temperature of 24.2, 22.2, and 19.9 °C for T11, T12 and T13, respectively. The coating S1 had no obvious phase change characteristics, and the S8 and S9 phase transition temperatures were not in the range of the surface temperature and the dew point temperature. S1, S8, and S9 have no obvious difference in anticondensation time. While the phase transition temperatures of S10, S11, and S12 were in the interval between the surface temperature and dew point temperature, the anticondensation performance is improved. The law of ascension was consistent with S4–S7, and as the phase change heat storage value increased, the anticondensation time increased. The S13 phase transition temperature range partially fell at 30–19.9 °C, so the anticondensation time had a certain increase but not as obvious as S12.

## 4. Discussion

Combined with the above analysis, it can be inferred that the phase change coating has anticondensation characteristics, which are based on the temperature control characteristics of the coating. That is, when the phase transition temperature is between the ambient temperature and the surface temperature, there is a “temperature platform” phenomenon during the coating temperature drop [15], which slows down the temperature drop, thereby reducing the temperature difference between the surface temperature and the dew point and slowing down the condensation. 

Therefore, according to different ambient temperatures and surface minimum temperatures, a suitable phase change coating can be selected so that the phase transition temperature point is in this interval, and the temperature control characteristic can be utilized to slow down the surface temperature drop speed, thereby slowing down the condensation. The larger the phase change is, the lower the thermal conductivity, the better the temperature control performance, and the more the anticondensation performance is improved.

## 5. Conclusions

Based on the advantages of anticondensation coating, this paper proposed a temperature-control coating based on phase change materials and deeply studied its application in anticondensation of ring network cabinets. It has been verified by simulation tests that the temperature-control coating can truly reduce the generation of condensation. Different types of composite coatings can exert anticondensation effects under different application conditions. Based on the layout of the cabinet, the material characteristics and the heat balance equation of the fluid and solid surface, the principle of anticondensation of the temperature-control coating was explored by means of a simulation test.

Phase change materials with different phase transition temperatures were added to liquid silicone rubber material, and composite materials with phase change characteristics were prepared. The physical and chemical properties of the composite materials are close to those of the liquid silicone rubber materials without the addition, but the thermal and electrical properties are significantly different. The phase change material introduces a phase change characteristic into the composite material, and a “temperature platform” appears. The phase transition temperature point of the composite material to which the phase change material is added increases due to the increase in the proportion of the filler and finally approaches the phase transition temperature point of the phase change material itself. The enthalpy heat storage in the composite phase transition process is related to the proportion of the filler. The larger the proportion of the phase change filler is, the higher the phase transition enthalpy of the composite.

## Figures and Tables

**Table 1:** Sample serial number and type comparison table.

Serial Number	Filler and Mass Fraction	Serial Number	Filler and Mass Fraction
S1	/	S10	10% F2
S2	2.5% F1	S11	20% F2
S3	5% F1	S12	30% F2
S4	10% F1	S13	40% F2
S5	20% F1	S14	2.5% F1, 2.5% F2
S6	30% F1	S15	5% F1,5% F2
S7	40% F1	S16	10% F1, 10% F2
S8	2.5% F2	S17	20% F1, 20% F2
S9	5% F2	/	/

**Table 2:** Condensation simulation test conditions setting.

Working Condition Number	Cabinet Temperature/°C	Relative humidity/RH%	Dew Temperature/°C	Surface Temperature/°C
T1	20	90	18.3	14.3
T2	20	80	16.4	12.4
T3	20	70	14.4	10.4
T4	25	90	23.2	19.2
T5	25	80	21.3	17.3
T6	25	70	19.1	15.1
T7	25	90	28.2	20.2
T8	25	90	28.2	21.2
T9	30	90	28.2	22.2
T10	30	90	28.2	23.2
T11	30	90	28.2	24.2
T12	30	80	26.2	22.2
T13	30	70	23.9	19.9
T14	35	90	33.1	29.1
T15	35	80	31.0	27.0
T16	35	70	28.7	24.7

**Table 3:** S1–S7 heat flow diagram inflection point table.

Serial Number	Heat Flow Rate Map Inflection Point (°C)	Phase Change Heat Storage (J/g)
S1	/	/
S2	/	/
S3	7.1	1.5
S4	12.4	3.4
S5	17.0	8.0
S6	17.9	11.9
S7	20.8	15.8

**Table 4:** S1 and S8–S13 heat flow diagram inflection point table.

Serial Number	Heat Flow Rate Map Inflection Point (°C)	Phase Change Heat Storage (J/g)
S1	/	/
S8	14.1	1.3
S9	19.2	2.7
S10	25.1	6.1
S11	26.2	9.2
S12	30.1	11.6
S13	32.3	16.2
